# Case analysis of complete remission of advanced hepatocellular carcinoma achieved with sorafenib

**DOI:** 10.1186/s40001-015-0085-9

**Published:** 2015-02-04

**Authors:** Daizhong Liu, Aixiang Liu, Junping Peng, Yong Hu, Xielin Feng

**Affiliations:** Department of Surgery, Sichuan Cancer Hospital, Chengdu, 610041 Sichuan Province China

**Keywords:** Advanced hepatocellular carcinoma, Sorafenib, Complete remission, Literature review

## Abstract

**Background:**

To evaluate the feasibility and security of complete remission (CR) of advanced hepatocellular carcinoma (HCC) achieved with sorafenib treatment, and investigate the previously described predictive factors in CR.

**Methods:**

The case of a patient who achieved CR of advanced HCC with sorafenib treatment was analyzed. The case analysis was performed by a literature review of relevant reports retrieved from the PubMed database.

**Results:**

A 58-year-old male patient achieved CR of advanced HCC after 23 weeks of oral treatment with sorafenib alone for 41 months and maintained CR for more than 35 months. Eleven reports worldwide have documented a total of twelve patients who achieved CR of advanced HCC, including six with nonsurgical oral sorafenib treatment, four with surgical resection in the descent stage following oral sorafenib treatment and two with oral sorafenib treatment for postoperative metastasis.

**Conclusions:**

For unresectable advanced HCC, sorafenib can significantly improve progression-free survival and overall survival, achieving CR in some cases. In addition, surgical resection of advanced HCC in the descent stage is possible following oral sorafenib treatment. For patients with postoperative distant metastasis of HCC, sorafenib treatment also provides clinical benefits and can even achieve CR. Besides, long-term sorafenib administration is safe, and patients should continually receive sorafenib to avoid recurrence after complete remission of cancer. Furthermore, early HFSR, rapid decline of AFP levels and rapid tumor shrinking observed by imaging are known parameters describing sorafenib’s effects. Finally, it is important to assess the gene locus of sorafenib sensitivity in HCC patients in future research.

## Background

Hepatocellular carcinoma (HCC) constitutes 90% of primary liver cancer (PLC), which is the fifth most common malignant tumor and the third major cause of cancer-related deaths worldwide. In recent years, the global incidence of HCC has gradually increased, with more than 620,000 new patients currently diagnosed yearly. However, only 20% of patients with HCC can receive curative local treatment such as surgical resection, liver transplantation, and radiofrequency ablation, among others. In addition, the vast majority of patients present to the clinic with advanced stage disease; these patients typically die within three to six months as a result of lack of effective treatment [1]. Consequently, advanced HCC is known for its poor prognosis.

Sorafenib is a small-molecule multikinase inhibitor that mainly inhibits kinases including Raf kinase, vascular endothelial growth factor receptor (VEGFR), and platelet-derived growth factor receptor (PDGFR). Two randomized, double-blinded, controlled large phase III clinical trials, the SHARP (Sorafenib HCC Assessment Randomized Protocol trial) and the Asia-Pacific (conducted in the Asia-Pacific region) trials clearly demonstrated that sorafenib is currently the only systemic agent, not only improving progression-free survival significantly, but also enhancing overall survival in patients with unresectable advanced HCC [2,3]. Unfortunately, a complete remission (CR) was not achieved in patients of the sorafenib group, indicating that achieving CR is rare after sorafenib treatment.

In this study, we present a case of advanced HCC associated with portal vein tumor thrombosis; the patient was treated with sorafenib alone for 41 months and achieved CR that was maintained for more than 35 months. Only 12 advanced HCC patients were previously described who achieved CR after sorafenib administration. To the best of our knowledge, the case reported here represents the longest documented sustained CR of primary liver lesions with sorafenib treatment; it is also the longest administration time recorded. The 12 previous cases of CR were assessed to explore the feasibility and security of CR and the previously described predictive factors.

## Case presentation

The patient was a 58-year-old male, who had fatigue and discomfort without obvious incentive in 1990. This patient had no other discomforts such as fever, vomiting, abdominal pain or diarrhea. Neither xanthochromia nor icteric sclera was detected. After being admitted into a local hospital, the patient was diagnosed with a hepatitis B virus (HBV) infection following detection of hepatitis B and elevated transaminase levels. His condition improved upon hospitalization and treatment. In 1997, the HBV marker was negative and liver function normal.

During a routine examination in 2008, this patient had an elevated alpha-fetoprotein (AFP, which is the specific tumor marker of HCC) level of 56 IU/ml (normal level 0 to 8 IU/ml). Computed tomography (CT) scans showed the presence of cirrhosis, splenomegaly, and a 0.9 cm × 0.8 cm small nodule of an undetermined nature in the right liver lobe. The patient was consequently given a diagnostic intervention of a focal liver lesion under local anesthesia by transcatheter hepatic arterial chemoembolization (TACE) on November 19, 2008 (drugs administered included: epirubicin, 20 mg; hydroxycamptothecine, 20 mg and ultra-fluid lipiodol, 3 ml). No positive marker was detected during the surgery and anti-inflammatory, liver-protective and symptomatic treatment was given postoperatively.

Four days after the surgery, the patient’s AFP level declined to 92.64 ng/ml (normal level 0 to 20 ng/ml). On January 13, 2009, the reexamination results revealed that the AFP level had slightly increased to 224.55 ng/ml. CT scans demonstrated the presence of a small 1.9 cm × 1.4 cm sheet-like low-density shallow in the left lobe of liver, the nature of which was yet to be determined. Contrast ultrasonography data indicated the presence of a hypoechoic nodule in the left liver, supporting the diagnosis of a small HCC. The patient consequently underwent radiofrequency ablation (RFA) surgery on January 19, 2009 (needle length, 4 cm; temperature, 105°C; voltage, 150 V; treatment time, 10 min). A month later, the AFP level dropped to a normal level (15.43 ng/ml).

During follow-up, the patient’s AFP level elevated to 29.25 ng/ml on April 22, 2009. Contrast ultrasonography data showed that the arterial phase on the top right of the primary tumor was significantly enhanced. As the disease can reoccur after the first RFA, the patient underwent a second RFA surgery on April 24, 2009 (needle length, 5 cm; temperature, 105°C; voltage, 150 V; treatment time, 10 min), followed by two TACE surgeries on May 4 and September 14, 2009, respectively (drugs administered included: epirubicin, 40 mg; hydroxycamptothecin (HCPT), 20 mg and ultra-fluid lipiodol, 10 ml). Postoperative follow-up data showed that the patient was disease free.

**Figure 1:**
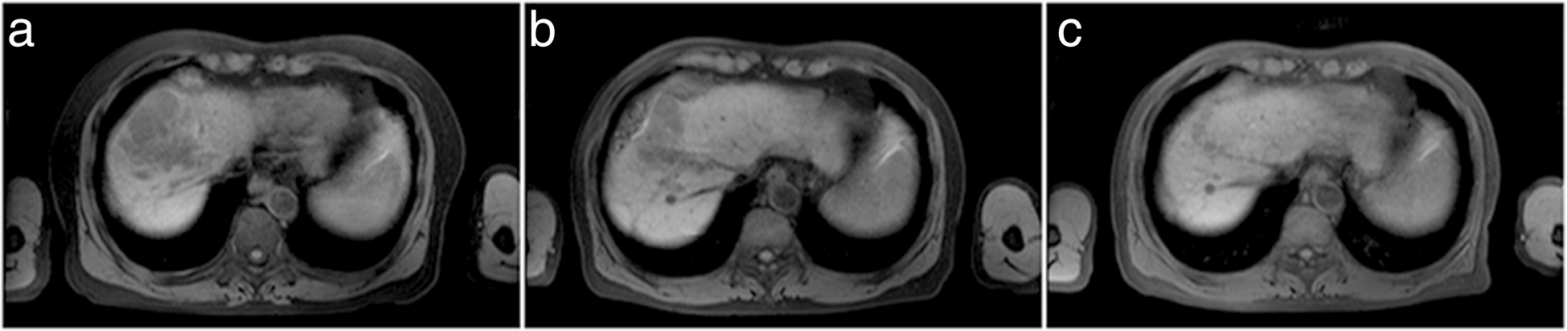
**The tumor shrank step by step during early sorafenib treatment. (a)** Before targeted therapy, tumor size was 7.7 cm × 7.9 cm × 6.3 cm; **(b)** week 5 of targeted therapy, tumor size decreased by 39.8%; **(c)** week 15 of targeted therapy, tumor size decreased by 96.9%.

On October 25, 2010, the patient was reexamined in Sichuan Cancer Hospital. The AFP level was found to be significantly high at 51.750 ng/ml. Magnetic resonance imaging (MRI) scans showed that the liver had multiple masses (maximal size, 5.4 cm × 7.9 cm) associated with portal vein tumor thrombosis (lesion fusion range, 7.7 cm × 7.9 cm × 6.3 cm; Figure 1a). The examination data from outpatient services showed that the patient’s physical condition was generally good (Eastern Cooperative Oncology Group (ECOG) score, 0), with no xanthochromia or icteric sclera, no positive signs of illness and no ascites. The blood coagulation function was normal, and liver function was ranked to Child-Pugh A (Child-Pugh is the grading standard of liver functional assessment, and for the choice of treatment plan provides an important reference). Accordingly, the patient was diagnosed with advanced HCC, classified as stage C of the Barcelona clinical liver cancer staging system.

From October 26, 2010, the patient received outpatient treatment of daily oral sorafenib. The initial dose of oral sorafenib was 800 mg/day, twice daily (morning and night). Three days later, the patient had a severe hand-foot skin reaction (HFSR) with persistent diarrhea (four to six times daily). As a result, the dose of oral sorafenib was adjusted to 400 mg/day (twice daily). The HFSR symptoms were treated with urea ointment, aloe cream, and emollient cream by local topical therapy. The diarrhea was treated by oral administration of smectite and loperamide hydrochloride The above symptoms were gradually alleviated after appropriate symptomatic treatments. The dose of oral sorafenib was adjusted to 600 mg/day (twice daily) from week 15 and then restored to the standard dose of 800 mg/day from week 23. A follow-up was conducted by strictly following the assistance project requirement of the China Charity Federation. The outpatient reexamination involved monthly examinations of AFP levels, liver function, and blood coagulation function, with bimonthly examinations by MRI.

**Figure 2:**
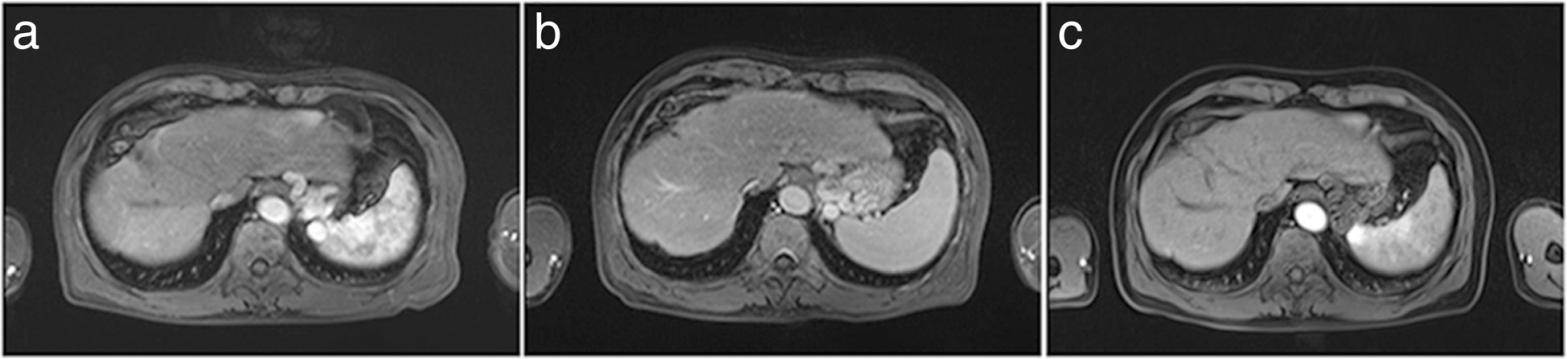
**Follow-up after complete remission achieved, there is no recurrence of tumor. (a)** Week 23 of targeted therapy, the tumor has completely disappeared; **(b)** year 2 of targeted therapy, the tumor has completely disappeared; **(c)** year 3 of targeted therapy, the tumor has completely disappeared.

Follow-up data showed that the HFSR and diarrhea were alleviated after oral sorafenib treatment for six months and the patient engaged in daily life and work during treatment. In week 5, MRI scans showed that the size of the tumor lesion declined by 39.8% to 7.5 cm × 5.4 cm × 5.7 cm; necrosis occurred in partial tissues of the tumor mass, as indicated by a clearer edge (Figure 1b); and AFP levels declined by more than 50%. In week 15, MRI scans showed that the size of tumor lesion declined by more than 96.9% to 2.8 cm × 1.7 cm × 2 cm; there was no tumor activity in the artery phase (Figure 1c); and the AFP level was close to the normal range, 54.33 ng/ml. In week 23, MRI scans showed that the tumor mass had completely disappeared (Figure 2a), and the AFP level had decreased to a normal level (Table 1). According the Response Evaluation Criteria In Solid Tumors (RECIST) criteria, the tumor examined by MRI had completely disappeared over four weeks, suggesting the achievement of CR.

**Table 1:** **Dynamic changes in alpha-fetoprotein (AFP) level of the patient with advanced hepatocellular carcinoma before and after sorafenib treatment**

**Detection time**	**Detection method**	**AFP (ng/ml)**	**Assessment**
2010.10.25	Chemiluminescence	51750.00	Normal range, 0–20 ng/ml
2010.11.29	Chemiluminescence	21668.00	Decrease by >50% in week 5
2011.02.16	Chemiluminescence	54.33	Close to normal in week 15
2011.04.14	Chemiluminescence	<10	Normal in week 23
2011.05.18–13.11.12	Chemiluminescence	<10	Normal for >30 months

To date, MRI examination shows that the tumor had completely disappeared in the patient over a period of 35 months. During follow-up, no radiographic evidence indicated the recurrence of HCC (Figure 2b,c), and the AFP level remained within the normal range. The patient was engaged in normal daily life and work and achieved sustained CR of advanced HCC.

### Literature review

Relevant reports were searched and retrieved manually from the PubMed database. Since the availability in 2007 of sorafenib for treatment of advanced HCC, a total of 11 reports worldwide have documented 12 patients who achieved CR of advanced HCC with this therapy [4-14] (Table 2).

**Table 2:** **Case summary of complete remission (CR) of advanced hepatocellular carcinoma achieved by sorafenib treatment**

**Case No.**	**Age (year), gender**	**Metastatic sites**	**Initial dose**	**CR time (month)**	**CR duration (month)**	**Publication time**	**Journal**	**Reference**	**Note**
1	78, male	Lung	400	5	6	17/10/08	*Journal of Hematology & Oncology*	So *et al.* [4]	First case of CR
2	54, male	Lung	400	18	Unknown	1/9/09	*American Journal of Transplantation*	Yeganeh *et al*. [5]	Solitary pulmonary metastasis post liver transplantation
3	59, male	Portal vein	400	6	16	14/3/10	*Liver International ISSN*	Irtan *et al*. [6]	Two cases reported in the same report; CR achieved by surgical resection in the descent stage following sorafenib treatment.
4	57, male	Portal vein	400	6	12	14/3/10	*Liver International ISSN*	Irtan *et al*. [6]	
5	74, male	Portal vein	200	8	16	23/3/10	*Targeted Oncology*	Wang *et al*. [7]	Initial dose at a low level
6	67, male	Portal lymph node	400	6	6	31/8/10	*Medical Oncology*	Chelis *et al*. [8]	HIV-hepatitis B virus (HBV) co-infected with advanced hepatocellular carcinoma
7	56, male	Thoracic diaphragm	400	6	6	20/4/11	*Journal of Clinical Oncology*	Curtit *et al*. [9]	CR achieved by surgical resection in the descent stage following sorafenib treatment.
8	84, male	Portal vein	400	6	6	11/4/11	*BMC Gastroenterology*	Sacco *et al*. [10]	First case of CR in hepatitis C patients in older people
9	76, female	Lung	200	4	8	22/12/11	*Oncology*	Inuzuka *et al*. [11]	First female case
10	60, male	Portal lymph node	400	11 days	12	24/7/12	*Oncology*	Mizukami *et al*. [12]	Shortest time to achieve CR
11	52, male	Lung	400	6	42	10/10/12	*Journal of Gastrointestinal Cancer*	Lulla *et al*. [13]	Longest duration of CR
12	66, male	Portal vein	400	6	8	15/2/13	*World Journal of Gastrointestinal Oncology*	Kim *et al*. [14]	

## Discussion

Sorafenib is a small-molecule multikinase inhibitor that primarily inhibits Raf kinase, VEGFR, and PDGFR. It is unique in targeting the Raf/Mek/Erk (MAP Kinase) pathway [15], as HCC mainly involves overexpression of the RAF/MEK/ERK pathway [16] In addition, extracellular activation of VEGFR and PDGFR are involved in the formation of HCC and portal vein tumor thrombosis. It is assumed that sorafenib has dual activity by both inhibiting tumor cell proliferation as well as tumor angiogenesis [17]. Two large stage III clinical trials, the SHARP trial and the Asia Pacific trial, reported that sorafenib administration increases the survival time of advanced HCC, however neither trial achieved CR in cases of advanced HCC [2,3].

During the literature search, only 11 reports were found worldwide that had documented 12 patients with CR of advanced HCC since the introduction of sorafenib for the treatment of advanced HCC in 2007. The twelve cases of CR included six with nonsurgical oral sorafenib treatment, four with surgical resection in the descent stage following oral sorafenib treatment [6,9,13] and two with oral sorafenib treatment for postoperative metastasis [5,12]. In the present study, the case of a 58-year-old male patient who achieved CR of advanced HCC associated with portal vein tumor thrombosis is reported. In this patient, CR was achieved after 23 weeks of oral sorafenib treatment; he was treated with sorafenib alone for a total of 41 months and maintained CR for more than 35 months. To the best of our knowledge, it is the longest documented sustained CR of primary liver lesions with sorafenib treatment; it is also the longest administration time recorded. The results of this case analysis demonstrate that for unresectable advanced HCC, sorafenib treatment can significantly improve progression-free survival and overall survival, achieving CR in some cases. In addition, sorafenib treatment enables surgical resection of the tumor lesion in the descent stage of advanced HCC. In cases of postoperative distant metastasis of HCC, administration of sorafenib also provides clinical benefits and can even achieve CR.

In the reports reviewed in this manuscript, the cases of CR lacked definite pathological diagnosis. Among the six patients with nonsurgical oral sorafenib treatment, only one was diagnosed as HCC by liver biopsy whilst the other five patients had no pathological diagnosis. Of these, two were diagnosed with HCC by positron emission tomography (PET)-CT, and the remaining three patients shared the following three common characteristics: (1) a history of hepatitis; (2) typical imaging findings of HCC; and (3) abnormally elevated AFP, which returned to normal after treatment with sorafenib. It is known that in the Asia Pacific, the majority of HCC patients have a background of liver cirrhosis, the specificity of typical imaging findings, combined with the criterion of AFP >400 ng/ml resulting in HCC diagnosis of close to 100% [18]. In the case reported in this present study (this 58-year-old male patient), both the continuous shrinking of tumor size in the MRI scans and the rapidly decreasing AFP levels during sorafenib treatment indicate that the CR of advanced HCC was induced by sorafenib. This case meets the diagnostic criteria for advanced HCC. For advanced HCC patients under nonsurgical oral sorafenib treatment, MRI imaging diagnosis combined with the criterion of AFP >400 ng/ml provides an objective indicator for HCC diagnosis and therapeutic assessment.

The most common adverse reactions of sorafenib comprise rash, diarrhea, HFSR, fatigue, and alopecia. Other adverse reactions include nausea and vomiting, anorexia, and hypertension [19]. However, these adverse reactions can be easily controlled, and patients seemed to well tolerate the drug in the SHARP and Asia Pacific trials [2,3]. Our patient had excellent adherence to the medication and did not terminate oral sorafenib even with severe HFSR and diarrhea. This patient continued sorafenib medication under appropriate symptomatic treatments. This is in contrast to the patients included in the SHARP trial, where the discontinuation rate of medication was up to 36% [2]. Most cases of CR were discontinued from taking sorafenib because of economic reasons and/or the drug’s adverse effects. However, it is unknown whether CR is maintained following discontinuation. The patient reported here continued sorafenib treatment after he had achieved CR. To date (March 25, 2014), around 41 treatment months have been sustained. His quality of life has not been affected and other indicators such as liver function are normal: he lives a normal life and works without problem. Therefore, long-term drug use seems safe, and HCC patients should continue to receive sorafenib after CR to avoid recurrence.

Studies show that early HFSR [20] and the rapid decline of AFP levels [21] are indicative of the antitumor activity of sorafenib, thus supporting the good therapeutic effects of sorafenib on HCC patients. These two parameters can therefore be used as early indicators for predicting the therapeutic effects of sorafenib treatment, which will help to adjust the treatment plan. Our patient had severe HFSR after three days of medication; meanwhile, his AFP level decreased rapidly to more than 50% in early treatment. These findings are supported by previous works. Not only that, MRI scans showed rapid shrinkage of the tumor size during early sorafenib treatment (39.8% in week 5 and by 96.9% in week 15), which indicated that rapid tumor shrinking on the imaging may be another indicator of the therapeutic effects of sorafenib.

HCC has unique molecular biological characteristics. Targets of sorafenib, including Raf kinase, VEGFR and PDGFR are not expressed on all HCC, which explains why CR of advanced HCC is rare after treatment with sorafenib. If the gene locus of sorafenib sensitivity in HCC could be assessed in HCCs, patients might be able to receive individualized treatments, which perhaps would lead to more individuals achieving CR. Although the lack of biopsy prevented us from exploring the case at the molecular level, we believe this case is of great value, providing evidence of individual cancer biology, which merits further investigation.

## Conclusions

In conclusion, for unresectable advanced HCC, sorafenib can significantly improve progression-free survival and overall survival, achieving CR in some cases. In addition, surgical resection of advanced HCC in the descent stage is possible following oral sorafenib treatment. For patients with postoperative distant metastasis of HCC, sorafenib treatment also provides clinical benefits and can even achieve CR. The adverse reactions of sorafenib are predictable. Patients might get clinical benefits and even achieve CR by adopting reasonable and accurate measures for the early sorafenib adverse reactions; this could increase patient’s willingness during treatment to continue sorafenib medication. We consider sorafenib very safe for long-term administration, which can prevent recurrence after CR. Furthermore, early HFSR, rapid decline of AFP levels and rapid tumor shrinking are the previously observed parameters of sorafenib’s curative effects. Finally, future research is necessary to assess the gene locus of sorafenib sensitivity in HCC.

### Consent

Written informed consent was obtained from the patient for the publication of this report and any accompanying images.

## References

[1] Shariff MI, Cox IJ, Gomaa AI, Khan SA, Gedroyc W, Taylor-Robinson SD (2009). Hepatocellular carcinoma: current trends in worldwide epidemiology, risk factors, diagnosis and therapeutics. Expert Rev Gastroenterol Hepatol..

[2] Llovet JM, Ricci S, Mazzaferro V, Hilgard P, Gane E, Blanc J-F (2008). Sorafenib in advanced hepatocellular carcinoma. N Engl J Med..

[3] Cheng AL, Kang YK, Chen Z, Tsao CJ, Qin S, Kim JS (2009). Efficacy and safety of sorafenib in patients in the Asia-Pacific region with advanced hepatocellular carcinoma: a phase III randomised, double-blind, placebo-controlled trial. Lancet Oncol..

[4] So BJ, Bekaii-Saab T, Bloomston MA, Patel T (2008). Complete clinical response of metastatic hepatocellular carcinoma to sorafenib in a patient with hemochromatosis: a case report. J Hematol Oncol..

[5] Yeganeh M, Finn RS, Saab S (2009). Apparent remission of a solitary metastatic pulmonary lesion in a liver transplant recipient treated with sorafenib. Am J Transplant..

[6] Irtan S, Chopin-Laly X, Ronot M, Faivre S, Paradis V, Belghiti J (2011). Complete regression of locally advanced hepatocellular carcinoma induced by sorafenib allowing curative resection. Liver Int..

[7] Wang SX, Byrnes A, Verma S, Pancoast JR, Rixe O (2010). Complete remission of unresectable hepatocellular carcinoma treated with reduced dose of sorafenib: a case report. Target Oncol..

[8] Chelis L, Ntinos N, Souftas V, Deftereos S, Xenidis N, Chamalidou E (2011). Complete response after sorafenib therapy for hepatocellular carcinoma in an HIV-HBV co infected patient: Possible synergy with HAART? A case report. Med Oncol..

[9] Curtit E, Thiery-Vuillemin A, Nguyen T, Heyd B, Pivot X, Di Martino V (2011). Complete histologic response induced by sorafenib in advanced hepatocellular carcinoma: a case report. J Clin Oncol..

[10] Sacco R, Bargellini I, Gianluigi G, Bertini M, Bozzi E, Altomare E (2011). Complete response for advanced liver cancer during sorafenib therapy: case report. BMC Gastroenterol..

[11] Inuzuka T, Nishikawa H, Sekikawa A, Takeda H, Henmi S, Sakamoto A (2011). Complete response of advanced hepatocellular carcinoma with multiple lung metastases treated with sorafenib: a case report. Oncology.

[12] Mizukami H, Kagawa T, Arase Y, Nakahara F, Tsuruya K, Anzai K (2012). Complete response after short-term sorafenib treatment in a patient with lymph node metastasis of hepatocellular carcinoma. Case Rep Oncol..

[13] Lulla PD, Brammer JE, Bandeali S, Lynch GR (2013). Sustained complete remission of metastatic hepatocellular carcinoma with single agent sorafenib. J Gastrointest Cancer..

[14] Kim MS, Jin YJ, Lee JW, Lee JI, Kim YS, Lee SY (2013). Complete remission of advanced hepatocellular carcinoma by sorafenib: a case report. World J Gastrointest Oncol..

[15] Liu L, Cao Y, Chen C, Zhang X, McNabola A, Wilkie D (2006). Sorafenib blocks the RAF/MEK/ERK pathway, inhibits tumor angiogenesis, and induces tumor cell apoptosis in hepatocellular carcinoma model PLC/PRF/5. Cancer Res..

[16] Yoshida T, Hisamoto T, Akiba J, Koga H, Nakamura K, Tokunaga Y (2006). Spreds, inhibitors of the Ras/ERK signal transduction, are dysregulated in human hepatocellular carcinoma and linked to the malignant phenotype of tumors. Oncogene..

[17] Novi M, Lauritano EC, Piscaglia AC, Barbaro B, Zocco MA, Pompili M (2009). Portal vein tumor thrombosis revascularization during sorafenib treatment for hepatocellular carcinoma. Am J Gastroenterol..

[18] Trevisani F, DIntino PE, Morselli-Labate AM, Mazzella G, Accogli E, Caraceni P (2001). Serum alpha-fetoprotein for diagnosis of hepatocellular carcinoma in patients with chronic liver disease:influence of HBsAg and anti-HCV status. J Hepatol.

[19] Grünwald V, Heinzer H, Fiedler W (2007). Managing side effects of angiogenesis inhibitors in renal cell carcinoma. Onkologie..

[20] Vincenzi B, Santini D, Russo A, Addeo R, Giuliani F, Montella L (2010). Early skin toxicity as a predictive factor for tumor control in hepatocellular carcinoma patients treated with sorafenib. Oncologist..

[21] Shao YY, Lin ZZ, Hsu C, Shen YC, Hsu CH, Cheng AL (2010). Early alpha-fetoprotein response predicts treatment efficacy of antiangiogenic systemic therapy in patients with advanced hepatocellular carcinoma. Cancer..

